# Proceedings of the Iowa State Medical Society

**Published:** 1867-12

**Authors:** 


### PROCEEDINGS OF THE IOWA STATE MEDICAL SOCIETY

The Iowa State Medical Society met at the Council Chamber, in Davenport, May 22d, 1867, at 10 o'clock A. M., the attendance being fair,

The President of the Society, Dr. J. W. H. Baker, took the chair and called the meeting to order.

At the request of the President, prayer was then offered by Rev. S. M. Anderson, of the Presbyterian Church.

Dr. T. J. Saunders, on behalf of the President of the Scott County Medical Society, Dr. T. J. Iles, whose health is impaired, received the delegates from abroad, in the following address of welcome:

### ADDRESS.

*Gentlemen of the State Medical Society:*

Upon this, our seventeenth anniversary, the duty devolves for the fourth time upon the fraternity of Davenport to extend to you the hand of cordial and sincere greeting.

If any there are in this assemblage, who, half a generation ago, had the honor of being at the first meeting of the State Medical Society of Iowa, the fact, probably, will be recognized, that a comparatively new set of actors occupy the stage, sadly reminding in the words of one of our gifted poets, that

"Art is long and Time is fleeting,
And our hearts though stout and brave;
Still, like muffled drums are beating
Funeral marches to the grave."

Though many devoted pilgrims in the ranks of our noble calling have stepped aside and are seen of men no more, yet, the Sience of Medicine remains, and with each succeeding year gathers unfaded laurels. Its votaries of the present day can be numbered by thousands, strong and united, enclosing and exercising as with a giant's power, a large share of the rapidly developing intellectuality of this most remarkable century. He who thinks we are only perpetuating a relic of antiquity, giving it neither vitality, nor form, nor comeliness, in accordance with the spirit of the age, is involved in worse than darkness, respecting our aims and accomplishments.

As living, active, energetic members of the profession, to us, in connection with our brethren throughout our wide-spread country, comes the duty of sustaining and advancing by all means at our command, that *prestige* of potency which has always attached to the regularly constituted guardians of life and health. With the spirit predominant, of each one casting in his mite, and full of trust that, like bread thrown upon the waters and gathered after many days, beneficial results to our organization shall be reached eventually, let us enter upon the transaction of such business as may come before us: and the hope is fervently entertained that, when the conclusion arrives, the remembrance of your stay with us may be accompanied by no emotion adverse to those of satisfaction and pleasure.

The Secretary, Dr. W. F. Peck, then read the minutes of the last regular meeting, which were approved, and the action of the President and Secretary, in calling the meeting at this time, was also approved.

The reading of the President's address was postponed until two o'clock P. M.

Dr. Peck moved that gentlemen present, members of the profession, yet not members of the Society, be invited to seats in the meeting, which was agreed to.

Dr. Bell moved that Students of Medicine be also invited to seats in the meeting, which was agreed to.

Dr. William Watson, of Dubuque, being the only member of the Board of Censors present, the Chair appointed to the vacancies, Dr. Thomas J. Saunders, and Dr. John Bell, of Davenport, Dr. Edward Whinery, of Fort Madison, and Dr. J. C. Hughes, of Keokuk.

On motion of Dr. Hughes, adjourned till 2 o'clock P. M.

---

AFTERNOON SESSION.

The Society met at 2 o'clock P. M., agreeably to adjournment.

Dr. Baker then delivered his annual address which proved to be upon "Medicine not an Exact Science." The discourse was liberal in its tone, full of striking and useful thoughts, and occupied a short half hour in the reading.

On motion it was resolved that the address be received and referred to the Committee on Publication.

Dr. Watson, from the Board of Censors, reported the following gentlemen for membership:

Dr. J. W. Graham, graduate of Jefferson Medical College, Pennsylvania, 1866-7.

Dr. J. C. Shrader, graduate of the Medical Department University of Iowa, 1864-5.

Dr. N. S. Smith, graduate Medical Department University of Iowa, 1863-4.

Dr. A. C. Moon, graduate of Albany Medical College, New York, 1842.

Dr. Edward H. Hazen, graduate of Medical College, Cleveland, Ohio, 1865-6.

Dr. M. Marbourg, graduate of Jefferson Medical College, Pennsylvania, 1859.

Dr. C. Roundy, graduate of Lind University, Chicago, Ills., 1861-2.

Dr. John J. Olshausen, graduate of Medical Department, Kiel, Holstein, 1847.

These gentlemen, upon being balloted for, were unanimously elected, respectively.

The Secretary read a communication from Dr. Cochran, Superintendent of the Orphan's Home, extending an invitation to visit the institution.

On motion of Dr. Hughes, the invitation was accepted, and the interval between the morning and afternoon sessions of Thursday, the 23d, agreed upon as the time for proceeding there.

Dr. M. B. Cochran, Treasurer, made a report, showing $218 50 in the Treasury. Report accepted.

The Chair made a report, stating the reason why the Committee on Publication had published no proceedings during the past year, was, that the amount of funds on hand did not warrant the Society's attempting to print the large mass of matter which had accumulated.

Dr. J. C. Hughes, from the Committee on Surgery, was called upon and made some very interesting statements in relation to a new mode of operating producing the elongation of a deformed bone, which was crowned with success. He also called attention to a case of his, wherein he encountered much difficulty, arising from the use of an imperfectly manufactured instrument, such as any physician, is liable to procure in purchasing, and advising care in selection.

Dr. J. C. Stone, from the committee on Criminal Abortion, read a most valuable paper on the subject under his charge, wherein the matter was treated not only ably and at considerable length, but upon an elevated basis, commending itself to the moral and conscientious practitioner.

The report was accepted and laid upon the table for future consideration.

Dr. Hughes offered a resolution that a committee of one from each county represented, be appointed to nominate officers for the ensuing year, and to report a place of meeting, which was adopted, and the following gentlemen were appointed by the Chair:

Drs. Hughes, of Lee; Watson, of Dubuque; Stone, of Des Moines; Early, of Johnson; Smith, of Marshall, and Tomson, of Scott.

Dr. Bell, with permission, reported a case of diabetes and specimens resulting from a chemical amalysis of the urine. The preparations were made by that accomplished chemist, Mr. H. A. Ridgely, of the firm of Harrison & Starks, and excited much curiosity as well as admiration.

The report was accepted, and referred to the Committee on Publication.

Dr. Watson, from the Censors, made a supplementary report, recommending for membership as follows: Dr. John W. Cowden, Graduate of Western Reserve College, O., 1855. Which was received, and, upon ballot, he was elected unanimously, and was placed upon the Committee on Nominations for the county of Jackson.

On motion of Dr. Witherwax, adjourned till 7½ o'clock in the evening.

EVENING SESSION.

At 7½ o'clock P. M., the meeting was called to order, the President in the chair.

Dr. Peck, from the Committee on Surgical Diseases, of Organs of Generation, produced an elaborate essay on Syphilis, exhaustive in its character, and specifying particularly his mode of treatment. The reading was marked by the closest attention on the part of the audience, and the impression was produced that the subject had been treated with unusual ability.

The report was accepted, and referred to the Committee on Publication.

Dr. Watson, from the Committee on Obstetrics, reported that he was not prepared to report at the present session. The committee was continued to report at next meeting.

Dr. Watson, from the Board of Censors, reported for membership as follows:

Dr. John S. Graham, Medical Department University of Iowa, 1849-50.

Dr. William Cornes, Medical Department, University of Iowa, 1863-4.

These gentlemen were balloted for and unanimously elected.

The report from the Committee on Urinary Calculi being called for

Dr. Whinery, in the absence of the chairman, read an interesting paper on the subject of an operation for Lithotomy, involving exceedingly novel results.

This statement called forth remarks from Dr. Hughes in relation to several interesting cases coming under his observation. Also from Drs. Cowden, of Bellevue, and Field, of Des Moines.

The report of Dr. Whinery was accepted, and referred to the Committee on Publication.

On motion, adjourned till 9 o'clock Thursday morning.

---

SECOND DAY.

At 9 o'clock A. M., the Society met, agreeably to adjournment, the President in the chair.

On motion, the reading of the minutes of yesterday's proceedings was dispensed with.

Dr. Hughes, from the committee on nominations, stated that they were ready to report, but desired a postponement until all the members should be present, which was agreed to.

The President laid before the meeting a communication from Dr. J. P. Gruwell, of Oskaloosa, giving his reasons at length for non-attendance, and evincing a commendable interest in the future of the Society.

Dr. Hughes, from the committee on nominations, then made a partial report, recommending that the Constitution be so amended as to make the place and time of meeting subject to the action of each annual meeting.

The report was received, and the recommendation unanimously adopted—this being necessary, constitutionally, in order to be operative.

Dr. Hughes, from the same committee, made a further report, that the next meeting be held at Des Moines, on the first Wednesday of February, which was adopted.

The committee also presented nominations for officers for the ensuing year, and the meeting proceeded to ballot, with the following result:

PRESIDENT—Dr. William Watson, of Dubuque.

VICE PRESIDENT—Dr. Edward Whinery, of Fort Madison.

RECORDING SECRETARY—Dr. A. G. Field, of Des Moines.

TREASURER—Dr. M. B. Cochran, of Davenport.

CORRESPONDING SECRETARY—Dr. E. J. B. Statler, of Marshalltown.

BOARD OF CENSORS—Dr. A. S. Maxwell, of Davenport; Dr. G. L. Carhart, of Mount Vernon; Dr. J. Williamson, of Ottumwa; Dr. A. M. Carpenter, of Keokuk; Dr. Fred. Lloyd, of Iowa City.

Drs. Cochran and Stone were appointed to conduct the newly elected President to the Chair, who, upon assuming the duties of the station, addressed the Society, briefly and gracefully, in acknowledgment of the honor conferred.

Dr. Field moved that a vote of thanks be tendered to Dr.

Baker, the retiring President, for the dignified and courteous manner in which he has conducted the proceedings during the past year, which was unanimously adopted.

Dr. Hughes moved that the thanks of the Society be also extended to the other retiring officers, and with the same result.

Dr. Early moved that the appointment of delegates to the American Medical Association be postponed until the meeting in February. Agreed to.

Various standing committees were called upon for reports and excuses for non-compliance rendered.

The committee on Epileptic Convulsions was continued.

Dr. Richardson, from the Committee on Meteorology and Medical Topography, read a paper upon the subject, treating of the matter generally, and containing a recommendation for the appointment of a committee with certain specified duties.

Upon this report an interesting discussion sprung up, in which Drs. Richardson, Hughes, Field, Watson, and Whinery participated.

On motion, the committee was continued, with authority to to follow the line recommended, and constituted as follows:

Dr. Richardson, of Davenport; Dr. Taylor, of Keokuk; Dr. Field, of Des Moines; Dr. Hudson, of Lyons; Dr. Statler, of Dubuque; Dr. Williamson, of Ottumwa; Dr. Harvey, of Burlington, and Dr. Shrader, of Iowa City.

On motion of Dr. Hughes, adjourned till 2 o'clock in the afternoon.

---

### AFTERNOON SESSION.

At the time adjourned to, the Society again met, the President, Dr. Watson, occupying the chair.

Several standing committees were called upon to report, and reasons were assigned for non-compliance.

Dr. Iles, from the standing committee on Cholera, presented a lengthy and able essay upon the subject, demonstrating that he had given the matter extensive research. Dr. Whinery then followed with an excellent paper upon the same topic, prepared and transmitted by Dr. Harvey, of Burlington.

The reports were accepted and ordered to be placed in the hands of the publishing committee.

The standing committee on Phthisis was continued. Also Committee on New Remedies. Also Committee on Necrology.

Dr. Maxwell, from the Committee on Hypodermic Injections, read a paper in relation thereto, prepared with much care and ability.

The report was received and referred to the Committee on Publication.

Dr. Cochran read a paper embodying the details of four cases of of *Cancrum Oris* occurring in his practice, and the treatment instituted therefor. The attention of the profession was particularly called to the fact that the disease is liable to be confounded with salivation, arising from excessive use of mercury. He stated that a recent instance of prosecution for mal-practice had been instituted against a respectable practitioner, by reason of evident misconception on this point.

The subject produced considerable remark—Drs. Lloyd, Cochran, Smith, Cowden and Bell participating.

Without disposing of the subject, and Dr. Bell having the floor, on motion, adjourned till 8 o'clock P. M.

---

THURSDAY EVENING SESSION.

At 8 o'clock P. M. the Society met, the President occupying the chair.

Dr. Hughes, with permission, offered the following resolution:

*Resolved*, That the State Medical Society take pleasure in adding their testimony to the prosperity, usefulness and management of the Soldiers' Orphans' Home at this place ; that, under the superintendence of Dr. Cochran and his able associates, the citizens of the State may congratulate themselves that the children under their care will receive every attention, and the interests of the State a watchful, properly guarded care ; and that the members of this Society tender their thanks to the Superintendent for his hospitable reception on the occasion of their visit to the institution this day.

Which having been read, the same was adopted unanimously.

Drs. Truesdale and Plummer, of Rock Island, being present, on motion, they were invited to participate in the proceedings.

The paper read by Dr. Cochran at the afternoon session, on *Cancrum Oris*, was accepted and referred to the committee on publication, without further comment.

Dr. Whinery, from the standing committee on Vaccination, made a report of considerable length, which was listened to with marked attention and interest. The report was received and referred to the committee on publication.

The Chair then announced the following standing committees:

*Surgery*—Drs. Baker, G. R. Henry and Hughes.

*Malignant Diseases*—Drs. Maxwell, Hughes and Graham.

*Diseases and Epidemics of Children*—Drs. Cochran, Ealy and Cleaver.

*Criminal Abortion*—Drs. Whinery, Thomson and Cornes.

*Medical Jurisprudence*—Drs. Huff, N. S. Smith and Baker.

*New Remedies*—Drs. Gamble, Knox and Shrader.

*Opthalmoscope and Diseases of the Eye*—Drs. Hughes, McClure and Saunders.

*Diabetes*—Drs. French, Bell and Gamble.

*Physical Exploration*—Drs. A. M. Carpenter, Harvey and Richardson.

*Urinary and Biliary Calculi*—Drs. Hudson, Cowden and Olshausen.

*Medical Inhalations*—Drs. McClure, J. W. Graham and Hudson.

*Cutaneous Diseases*—Drs. M. K. Taylor, Peck and Thrall.

*Uses and Abuses of Mercury*—Drs. Lloyd, Marbourg and Witherwax.

*Surgery of the Pelvic Organs*—Drs. Peck, Stone and Shrader.

*Sulphites and Hypo-Sulphites*—Drs. Rousseau, Hazen and Guthrie.

*Hypodermic Injections*—Drs. Gamble, Cleaver and Nassau.

*Use of New Instruments, and Devices for Diagnostic Purposes*—Drs. Field, Ealy and Blackburn.

Dr. Hudson, from the standing committee on Anæsthetics made a report, in which the respective merits of chloroform and æther, as agents, were ably discussed; also indicating the treatment, in case of threatening nervous depression arising from the use of anæsthetics.

Interesting remarks were made on the foregoing subject by Drs. Field and Hughes.

The report was received, and referred to the committee on publication.

The standing committee on cholera was continued as constituted last year.

Dr. W. H. Hosford was substituted for Dr. Sears, on the the Committee on Phitisis Pulmonalis; and Dr. Whinery was added to the Committee on Obstetrics.

Miscellaneous business now being regularly in order, Dr. Peck offered the following, which was adopted:

*Resolved*, That the committee on Publication be requested to confer with Dr. Hughes in relation to the practicability of publishing the proceedings of the Iowa State Medical Society, and that providing a reasonable contract can be agreed upon, the committee are authorized to appropriate the requisite amount from the treasury of the Society, not exceeding one hundred and twenty-five dollars.

The Chairman then announced as follows:

*Committee on Publication*—Drs. Field, Williamson and Carpenter.

The following resolution was offered and adopted:

*Resolved*, That a committee of three be appointed to report in detail to this Society, such subjects as they may deem of importance, for action at its next meeting.

Drs. Saunders, Hughes and Watson were appointed said committee.

A bill presented by Dr. Peck, late Secretary, for postage, &c., for $25 40, was ordered to be paid by the Treasurer.

Dr. Cochran offered the following resolutions, which were adopted:

*Resolved*, That the Polk County Medical Society be invited as a sister organization, to act as a committee of arrangements to supply accommodations for the next session of the Iowa

State Medical Society, which will be held in Des Moines, on the first Wednesday of February, 1868.

*Resolved*, That the Secretary of the Iowa State Medical Society, be instructed to address a copy of the above resolution to the President of the Polk County Medical Society.

A communication was received throwing doubt upon the claim of Dr. Miller, of Bellevue, to membership.

On motion, the matter was referred to a committee of three, consisting of Drs. Hughes, Hudson and Witherwax, to report at next meeting.

Dr. Lloyd offered the following resolutions, which were adopted:

*Resolved*, That the thanks of this Society are due, and are hereby tendered, to the members of the Scott County Medical Society, for the attention and hospitality which has rendered the present session of the State Society one of unmarred gratification and interest; and that the example set by our brethren of Davenport in ministering to our intellectual tastes and professional interest, as well as to our social enjoyments, is highly commendable and greatly appreciated.

*Resolved*, That our thanks are also tendered to the editors and reporters of the Davenport *Gazette* and Davenport *Democrat*, for furnishing, gratuitously, copies of their respective papers, containing the minutes of our proceedings, and for the completeness and correctness with which our transactions have been noted.

On motion, the thanks of the Society were tendered the City Council of Davenport for the use of their Chamber during the session.

On motion, the society then adjourned to meet in Des Moines, the first Wednesday in February, 1868. Feb 5,

---

WE WILL take this place to call the attention of the members of our State Society to the importance of a full attendance at our next meeting, at Des Moines, in February. Let the Profession of the State be present; and let not the committees whose names are presented, fail to make full and interesting reports on the various subjects which have been assigned them.

